# The GALA project: practical recommendations for the use of migalastat in clinical practice on the basis of a structured survey among Italian experts

**DOI:** 10.1186/s13023-020-1318-8

**Published:** 2020-04-07

**Authors:** Cristina Chimenti, Patrizia Nencini, Federico Pieruzzi, Sandro Feriozzi, Renzo Mignani, Maurizio Pieroni, Antonio Pisani, on behalf of the GALA Working Group, Yuri Battaglia, Anna Bersano, Agata Fiumara, Chiara Lanzillo, Stefania Piga, Federica Re, Marta Rubino, Maria Luisa Zedde

**Affiliations:** 1 Department of Cardiovascular, Respiratory, Nephrologic, Anesthesiologic and Geriatric Sciences, La Sapienza University of Rome, Viale del Policlinico 155, 00161 Rome, Italy; 2 Stroke Unit, Careggi University Hospital, Florence, Italy; 3 Clinical Nephrology, School of Medicine, University of Milano Bicocca, Milan, Italy; 4 Nephrology and Dialysis Unit, ASST-Monza San Gerardo Hospital, Monza, Italy; 5 Nephrology and Dialysis, Belcolle Hospital, Viterbo, Italy; 6 Department of Nephrology, Infermi Hospital, Rimini, Italy; 7 Cardiovascular Department, San Donato Hospital, Arezzo, Italy; 8 Department of Public Health, Nephrology Unit, University of Naples “Federico II”, Naples, Italy

**Keywords:** Fabry disease, Migalastat, Treatment, Enzyme-replacement therapy, Expert opinion

## Abstract

**Background:**

Oral migalastat has recently been approved for the treatment of Anderson-Fabry disease (FD) in patients aged ≥16 years with amenable mutations on the basis of two phase III trials, FACETS and ATTRACT. However, with the introduction of migalastat into clinical practice, it is important to correctly identify the patients who may gain the most benefits from this therapy. Due to the relatively recent availability of migalastat, its role in clinical practice still has to be included in guidelines or recommendations. On these bases, a multidisciplinary group of Italian Experts in the treatment of FD has run the GALA project, with the aim to collect the opinions of expert physicians and to propose some starting points for an experience-based use of migalastat.

**Results:**

Overall, although studies and data from longer-term follow-up with migalastat are still emerging, available evidence is consistent in showing that this molecule does represent a suitable therapy for the treatment of FD, in patients aged ≥16 years and with amenable mutations. The use of migalastat as an oral option appears to be overall safe, and experience thus far indicates potential for improving quality of life, controlling GI symptoms, stabilizing renal function and reducing cardiac hypertrophy.

**Conclusion:**

Migalastat can be considered either as a first-line therapy – given its efficacy, extensive tissue penetration, convenient oral regimen, and the current limited therapeutic options available – or in patients on enzyme-replacement therapy (ERT) who experience side effects, with poor compliance to chronic i.v. therapy, or with clinical evidence of progression of the disease.

## Background

Anderson-Fabry disease (FD) is a X-linked lysosomal disorder, due to deficiency of the enzyme α-galactosidase caused by mutations in the *GLA* gene (located on the long arm of the X chromosome at Xq22). This deficiency leads to the progressive accumulation of lysosomal glycosphingolipids, particularly globotriaosylceramide (GL-3) [1–4]. The accumulation of these substrates causes multiorgan damage and may eventually lead to major complications, including end-stage renal disease, hypertrophic cardiomyopathy and cerebrovascular events, with an increased risk of premature death [3, 4]. Cardiac arrhythmias, including conduction abnormalities, supraventricular and ventricular tachyarrhythmias can be the first clinical manifestation of the disease and can occur also in absence of hypertrophic cardiomyopathy [5].

FD is a relatively rare condition. Indeed, neonatal screening programs have reported with varying incidences of this condition, ranging from 1/1250 to 1/7800 in newborn males, although it has been observed that the later-onset phenotype of FD is underdiagnosed, making the incidence of a-Gal A deficiency 15–20-times higher than previously estimated [1, 6–8]. As FD causes tissue damage in different organs and systems, the therapeutic approach to patients with this condition should ideally be multidisciplinary and integrated within an individualized plan [4, 9]. Early initiation of therapy is crucial and contributes to optimize clinical outcomes [4]. Enzyme replacement therapy (ERT) has been approved in the treatment of FD since 2001 [10]. This approach also remains effective over a long-term period [4]; however, it is associated with some potential drawbacks including incomplete tissue penetration and reduced compliance due to a complex administration scheme [1, 11, 12]. Lastly, ERT likely has no proven effect on stroke and white matter lesion occurrence [13].

Migalastat (1-deoxygalactonojirimycin) is an analogue of the terminal galactose of GL-3; it is a pharmacological chaperone that stabilizes and facilitates trafficking of amenable mutant forms of the α-galactosidase A enzyme from the endoplasmic reticulum to lysosomes; in this site, dissociation of migalastat allows α-galactosidase to catabolize accumulated substrates [14, 15].

Oral migalastat administration has recently been approved for the treatment of FD in patients aged ≥16 years with amenable mutations on the basis of two phase III trials, FACETS and ATTRACT [15–17]. With the introduction of migalastat into clinical practice, it is important to correctly identify the patients who may gain the most benefits [4].

Due to the relatively recent availability of migalastat, its role in clinical practice has only been included in Canadian guidelines and is still to be considered for European recommendations [18].

On these bases, a multidisciplinary group of Italian Experts in the treatment of FD has run the GALA project, with the aim to collect the opinions of expert physicians and to propose an experience-based use of migalastat.

## The GALA project: overall architecture

The GALA project was initiated by a group of expert physicians on the treatment of FD and consisted of three different phases. In the first phase, the Expert Panel, composed of seven Experts from different backgrounds (cardiology, nephrology, neurology) with well-documented experience in the management of FD, as assessed by a number of international peer-reviewed publications, gathered and, with the help of a professional facilitator, elaborated a questionnaire aimed to evaluate the degree of consensus on the diagnosis and management of FD. The questionnaire was then administered to other Italian clinicians (*n* = 20, including the members of the GALA Working Group) with wide experience in the treatment of FD. The responses to the questionnaire were collected by an online system and analyzed by a dedicated provider (Springer Healthcare).

The results were then shared and discussed during a second meeting, where both the Expert Panel and the Working Group were present. The results were extensively discussed with the help of the professional facilitator. Experience-based recommendations on the use of migalastat were retrieved from the statements and reformulated until a consensus was reached.

### The questionnaire

The questionnaire consisted of two different sections, namely: (i) a descriptive section (12 items), aimed at describing the management of FD, ERT, and migalastat; and (ii) an analytic section (20 items), which evaluated migalastat therapy in different clinical scenarios. This latter section represented the basis for the definition of the recommendations. The questionnaire is reported in Table 1.

**Table 1:** The questionnaire on the management of Fabry disease (FD) (questions #1–12) and the use of migalastat (questions #13–32), and levels of agreement, expressed as percentages (*n* = 20)

Question number	Question	Disagreement (disagree + strongly disagree)	Neither agreement or disagreement	Agreement (agree + strongly agree)
1	The therapeutic goal for FD is the prevention and/or stabilization of organ damage	0	0	100
2	In a patient with FD and progressive organ damage, the main therapeutic goal is the control of symptoms and the long-term stabilization of clinical status	0	0	100
3	The impact on QoL is an important parameter in the management of a patient with FD	0	0	100
4	ERT is effective on most symptoms in FD	5	25	70
5	ERT is effective on organ damage in FD	10	20	70
6	Administration of ERT by i.v. route can be a limitation to daily activities in patients with FD	0	15	85
7	Potential immunogenicity of therapy can represent a limitation of ERT	0	20	80
8	Early initiation of treatment could improve the prognosis of patients with FD	0	5	95
9	When compared with i.v., oral therapy can improve QoL in patients with FD	0	5	95
10	According to available data, migalastat can be considered a safe and effective treatment for FD	0	0	100
11	According to available evidence, one of the advantages of migalastat over ERT is its superior efficacy on heart damage	0	30	70
12	Poor compliance to oral therapy with migalastat can be an issue	35	35	30
13	Migalastat therapy can be taken into consideration as an alternative to ERT in patients with FD and amenable mutations	0	0	100
14	In a male patient aged ≥16 years with amenable mutations and classic FD, migalastat therapy might be taken into consideration at diagnosis, even when signs/symptoms of organ damage are lacking	20	15	65
15	In a male patient aged ≥16 years with amenable mutations and classic FD, migalastat therapy is recommended in the presence of signs/symptoms of organ damage	5	15	80
16	In a male patient aged ≥16 years with amenable mutations and non-classic FD, migalastat therapy is recommended in the presence of signs/symptoms of organ damage	0	15	85
17	In a female patient aged ≥16 years with amenable mutations and classic FD, migalastat therapy might be taken into consideration at diagnosis, even when signs/symptoms of organ damage are lacking	45	20	35
18	In a female patient aged ≥16 years with amenable mutations and classic FD, migalastat therapy is recommended in the presence of signs/symptoms of organ damage	0	10	90
19	In a female patient aged ≥16 years, amenable mutation and non-classic FD, migalastat therapy could be taken into consideration at the first onset of signs/symptoms of organ damage	0	0	100
20	Migalastat therapy is recommended in patients with FD aged ≥16 years, with amenable mutations and heart hypertrophy (≥11 mm)	0	5	95
21	Migalastat treatment should be taken into consideration in patients aged ≥16 years with FD, amenable mutations and rhythm disorders (sinus bradycardia, atrial fibrillation, extrasystole) and/or ECG alterations	0	20	80
22	Migalastat therapy is recommended in patients with FD aged ≥16 years with amenable mutations and pathological microalbuminuria (according to KDIGO guidelines)	0	10	90
23	Migalastat therapy is recommended in patients with FD aged ≥16 years with amenable mutations and proteinuria (according to KDIGO guidelines)	0	10	90
24	Migalastat therapy is recommended in patients aged ≥16 years with FD, amenable mutations and eGFR 60–90 ml/min/1,73m^2^ (CKD-EPI) with evidence of progression of decline of renal function (> −1 ml/min/1,73m^2^/year)	0	15	85
25	Migalastat therapy is recommended in patients aged ≥16 years with FD, amenable mutations and eGFR 30–60 ml/min/1,73m^2^ (CKD-EPI)	0	20	80
26	Migalastat treatment could be taken into consideration in patients aged ≥16 years with FD, amenable mutations and progression of white matter lesions	5	10	85
27	Migalastat treatment should be taken into consideration in patients aged ≥16 years with FD, amenable mutations and history of TIA/stroke	0	10	90
28	Migalastat treatment should be taken into consideration in patients aged ≥16 years with FD, amenable mutations and progressive loss of hearing (corrected by age)	10	10	80
29	Migalastat treatment should be taken into consideration in patients aged ≥16 years with FD, amenable mutations and gastrointestinal symptoms	0	20	80
30	Migalastat treatment should be taken into consideration in patients aged ≥16 years with FD, amenable mutations and acroparesthesia, even if controlled by symptomatic therapy	5	15	80
31	In a patient aged ≥16 years and amenable mutations, already in therapy with ERT, switching to migalastat should be taken into consideration in the case of unstable disease and/or poor response	0	10	90
32	In a patient aged ≥16 years and amenable mutations, already in therapy with ERT, switching to migalastat should be taken into consideration in the case of uncontrolled infusion reactions and/or poor compliance to i.v. therapy	0	0	100

*eGFR* estimated glomerular filtration rate, *ECG* electrocardiogram, *ERT* enzyme replacement therapy, *FD* Fabry disease, *i.v.* intravenous, *KDIGO* Kidney Disease Improving Global Outcomes, *QoL* quality of life, *TIA* transient ischemic attack

Each item of the questionnaire was presented to define the degree of agreement on a precise statement, according to a 5-point Likert scale (1 = strongly disagrees, 2 = disagrees, 3 = neither agrees or disagrees, 4 = agrees, 5 = strongly agrees). In the analysis of data, the threshold for consensus was set at 75% of responders who agreed or strongly agreed upon a statement.

## Overview of the results

All the invited experts answered all items of the questionnaire. Table 1 summarizes the results of the voting: overall, a consensus was reached on the majority of items (26/32). A comment on each single item of the questionnaire goes beyond the scope of this article.

## The experience-based recommendations

The replies to the analytical phase were used to draft ten experience-based recommendations, which were then grouped according to the scenario they depict. Those statements are listed in Table 2 and commented in the following paragraphs.

**Table 2:** Expert-based recommendations on the use of migalastat in Fabry disease (FD)

According to current evidence, migalastat is an effective and generally well tolerated treatment for FD in patients with amenable pathogenic mutations.	
The use of oral therapy with migalastat can improve the quality of life of patients with FD.	
In a male patient aged ≥16 years with amenable mutations and type 1 classic FD, migalastat may also be considered at diagnosis when signs/symptoms of organ damage are not present.	
In a male patient aged ≥16 years with amenable mutations and type 2 late-onset FD, migalastat may also be considered at diagnosis at the presence of signs and symptoms of organ damage.	
In a female patient aged ≥16 years, with amenable mutations and type 1 classic or type 2 late-onset FD, migalastat can be considered at the presence of early signs/symptoms of organ involvement.	
Treatment with migalastat can be considered in patients with FD aged ≥16 years with amenable mutations, and heart hypertrophy and/or rhythm alterations and/or ECG alterations.	
Treatment with migalastat can be considered in patients with FD aged ≥16 years with amenable mutations and persistent microalbuminuria, and/or proteinuria and/or eGFR 30–90 ml/min/1.73m^2^	
Treatment with migalastat can be considered in patients with FD aged ≥16 years with amenable mutations and transient ischemic attack /stroke and/or white matter lesions.	
Treatment with migalastat can be considered in patients with FD aged ≥16 years with amenable mutations with acroparaesthesia, and/or gastrointestinal symptoms, and/or hearing loss.	
In a patient aged ≥16 years with amenable mutation already in treatment with ERT, switching to migalastat should be considered in the case of unstable patients and/or uncontrolled infusion reactions and/or poor compliance to intravenous therapy.	

*eGFR* estimated glomerular filtration rate, *ERT* enzyme replacement therapy, *FD* Fabry disease

### Migalastat: role in the therapy of FD

**Table:** 

• According to current evidence, migalastat is an effective and generally well tolerated treatment for FD in patients with amenable pathogenic mutations.	
• The use of oral therapy with migalastat can improve the quality of life of patients with FD.	
• In a male patient aged ≥16 years with amenable mutations and type 1 classic FD, migalastat may also be considered at diagnosis when signs/symptoms of organ damage are not present.	
• In a male patient aged ≥16 years with amenable mutations and type 2 late-onset FD, migalastat may also be considered at diagnosis at the presence of signs and symptoms of organ damage.	
• In a female patient aged ≥16 years, with amenable mutations and type 1 classic or type 2 late-onset FD, migalastat can be considered at the presence of early signs/symptoms of organ involvement.	

Migalastat is a small molecule pharmacologic chaperone with broad tissue distribution in multiple organs. It binds selectively and reversibly to the active sites of amenable mutant forms of α-galactosidase A enzyme. This high-affinity binding allows migalastat to stabilize the enzyme in the endoplasmic reticulum and facilitate proper trafficking to lysosomes. Once in lysosomes, migalastat dissociates from α-galactosidase A, allowing the breakdown of substrates [1].

A preliminary experience with migalastat is worth mentioning. In a pooled analysis of two phase II studies in males with FD, migalastat decreased urinary GL-3 by ≥20% in five out of six patients with amenable mutations, and serum levels remained overall unchanged [19]. GL-3 content was decreased in renal biopsies of four patients and skin biopsies of three patients with amenable mutations.

The clinical efficacy of migalastat has then been evaluated in the two pivotal phase III trials. The randomized, double-blind, placebo controlled, phase III FACETS study was conducted in ERT-naïve males and females (aged 16–74 years) with FD and amenable mutations [17]. Patients were randomly assigned to receive either migalastat 150 mg every other day (*n* = 34) or placebo (*n* = 33) for 6 months, followed by open-label migalastat for a further 6 months; patients could then participate in a 12-month open-label extension. Among the randomized patients, 50 (74.6%) were considered as amenable to migalastat based on the validated GLP HEK (Good Laboratory Practice human embryonic kidney) assay, which identifies GLA variants with the potential to respond to migalastat [20]. A 3.0% wild-type absolute increase was required based on literature indicating that increases of 1 to 5% of normal enzyme activity in vivo may be clinically meaningful [16].

These patients were included in the modified intent-to-treat (mITT) population used for post hoc efficacy analyses. While, with the ITT analysis, a numerically higher response rate was observed in the proportion of patients with ≥50% reduction in number of GL-3 inclusions per kidney interstitial capillary at 6 months (primary endpoint) with migalastat compared to placebo, the post hoc analysis on the mITT population demonstrated that migalastat was associated with a significantly greater reduction in the mean number of KIC GL-3 inclusions compared to placebo (− 0.25 ± 0.10 vs 0.07 ± 0.13; *p* = 0.008). In addition, the KIC GL-3 reduction remained stable for a further 6 months of treatment in the migalastat group, and the number of inclusions was reduced from 6 to 12 months in patients who switched from placebo to migalastat (− 0.33 ± 0.15 vs 0.01 ± 0.04; *p* = 0.01).

On the other hand, the randomized, open-label, phase III ATTRACT study included ERT-experienced patients aged 16–74 years, with FD and amenable mutation as assessed by the GLP HEK assay [15]. Patients were assigned to either continuation of ERT (*n* = 19) or switching to migalastat 150 mg QOD (*n* = 34) for 18 months; patients could then receive open-label migalastat for 12 months. Overall, migalastat and ERT had comparable effects on renal function: the mean annualised eGFR_CKD-EPI_ from baseline to month 18 was − 0.40 ± 0.93 (− 2.27 to 1.48) mL/min/1.73 m^2^/year for migalastat vs. − 1.03 ± 1.29 (− 3.64 to 1.58) mL/min/1.73 m^2^/year for ERT. The proportion of patients who experienced renal, cardiac, or cerebrovascular events was also similar with migalastat and ERT (29% vs 44%; *p* = 0.36). Patient-reported scores on the Brief Pain Inventory Short-Form-Pain Severity Component remained stable in both groups.

The baseline assessment of disease severity in ATTRACT was comparable with that reported for the Fabry Outcomes Survey (FOS) and Fabry Registry, and the majority of subjects had a classical phenotype, so that data from ATTRACT may be indirectly compared to results of clinical trials on ERT [21–23]. In addition, by evaluating patients in the FACETS trial by phenotype, it was observed that migalastat provided clinical benefit to patients with Fabry disease and amenable variants, regardless of disease severity [24].

We also mention that, as further discussed in the following paragraphs, data from a real-life study found that the renal function decline was not changed by 1-year treatment with migalastat [25]. So, patients started on oral therapy as first-line treatment should be followed with extra care as to detect poor renal response.

With respect to safety, in the FACETS study, most adverse events (AEs) were mild-to-moderate. The most frequently reported AEs with migalastat during the first 6 months of treatment were headache (35%) and nasopharyngitis (18%); the incidence of headache decreased to 14% during months 6–12. No serious migalastat-related AEs were reported [17]. After 18 months of treatment in the ATTRACT trial, the most frequently reported treatment-emergent AEs with migalastat were nasopharyngitis (33%) and headache (25%); these events occurred in 33 and 24% of patients assigned to ERT, respectively. No serious AE was considered to be migalastat-related [15].

Based on these results, migalastat was approved for the treatment of FD in patients aged ≥16 years with amenable mutations, and is thought to have potential for an improvement of patients management. Indeed, it has been shown that the effectiveness of ERT in preventing renal, cardiac and neurologic complications is limited, and patients tend to show signs of disease progression over time [26]. Thus, effective treatment options, such as migalastat, appear crucial to address this unmet need. In particular, several features of migalastat could make this molecule an attractive treatment option. First, oral administration may be more attractive for the majority of patients compared to the intravenous way of administration of ERT. Moreover, the migalastat dosing schedule has been shown to provide a sustained increase of α-galactosidase A level and migalastat is not expected to lead to the formation of anti-agalsidase antibodies [19]. Both of these attributes could increase treatment efficacy. Lastly, migalastat is expected, according to current and emerging evidence, to have better effects on cardiac outcomes and gastrointestinal symptoms than ERT, potentially improving both patient morbidity and quality of life. It should be acknowledged that further studies are required to investigate the long-term benefits of migalastat therapy, while some evidence is present for sustained activity of ERT to slow the decline in estimated glomerular filtration rate, and reduce/stabilize left ventricular mass and cardiac wall thickness [27].

### Patients with cardiac involvement

**Table:** 

Treatment with migalastat can be considered in patients with FD aged ≥16 years with amenable mutations, and heart hypertrophy and/or rhythm alterations and/or ECG alterations.	

Cardiac involvement in FD is common both in homozygous males and in heterozygous females and contributes substantially to disease-related morbidity and mortality. Moreover, the heart can be mainly involved in late-onset disease with specific genetic variants associated with residual enzymatic activity [28].

Cell GB3 accumulation leads to myocardial ischemia, valvular abnormalities, conduction tissue disease, arrhythmias, and myocardial hypertrophy. In particular, left ventricular hypertrophy (LVH) mimics the morphological and clinical picture of hypertrophic cardiomyopathy [29, 30], with early diastolic dysfunction and preserved ejection fraction until the end stage of the disease. Cardiac involvement characterized by early myocardial sphingolipid storage can be timely detected before overt LVH by cardiac MRI with T1 mapping [31].

In patients with FD, therapeutic goals are to reduce morbidity and mortality related to cardiac complications by early treatment with disease-specific therapies and conventional supportive therapy according to general cardiologic guidelines [4].

In the FACETS trial, a significant decrease in mean LVMi was observed from baseline up to 24 months (− 7.7 g/m^2^; 95% CI: − 15.4 to − 0.01), with a trend towards further reduction in patients with LVH at baseline [17]. A decrease in the end-diastolic interventricular septum thickness was also observed. A recent analysis of FACETS trial data dividing male patients in two subgroups (“classic phenotype” and “other patients”) showed that migalastat led to reductions in LVMi in both subgroups. In particular change from baseline to month 24 in LVMi was − 16.7 (SD 18.64) g/m^2^ (95% CI: − 31.1 to − 2.4; *n* = 9) in males with the classic phenotype and − 3.2 (18.66) g/m^2^ (95% CI: − 12.5–6.1; *n* = 18) in other patients [24]. In the ATTRACT trial, LVMi was significantly decreased from baseline at month 18 with migalastat (− 6.6 g/m^2^; 95% CI: − 11.0 to − 2.2) but not with ERT (− 2.0 g/m^2^; 95% CI: − 11.0–7.0) [15]. The largest LVMi changes with migalastat were observed in the subgroup of patients presenting baseline LVH.

On this basis, male and female patients aged ≥16 years with cardiac signs/symptoms of FD consisting of electrocardiographic [32] and/or echocardiographic and cardiac MRI evidence of LVH [5] and/or rhythm (brady and/or tachyarrhythmias) and ECG alterations (PQ interval < 120 ms, atrioventricular block, sinus node dysfunction, intraventricular conduction delay) [33] can be considered for the treatment with migalastat.

Nevertheless, up to now there is no evidence that treatment with migalastat may affect ECG alterations and arrhythmic manifestation of FD. Indeed, as myocardial fibrosis appears to be the major contributor of cardiac arrhythmias, it is unlikely that both ERT and migalastat will be effective in their treatment.

### Patients with renal involvement

**Table:** 

Treatment with migalastat can be considered in patients with FD aged ≥16 years with amenable mutations and persistent microalbuminuria, and/or proteinuria and/or eGFR> 30–90 ml/min/1.73 m^2^.	

Renal manifestations of FD occur early in life and if not treated progress to end-stage renal disease in nearly all male patients and some female patients [34]. Proteinuria is strongly associated with renal disease progression. Indeed, renal complications are key contributors to the morbidity and mortality associated with FD. In routine clinical practice, proteinuria and microalbuminuria are considered the earliest signs of FD nephropathy [34, 35].

Renal biopsy remains the hall mark of FD nephropathy, as significant GB3 accumulation has been found in several types of kidney cells especially in the podocytes, even in patients without overt signs of clinical kidney disease. An important finding was the detection of early segmental podocyte foot process effacement in most normoalbuminuric young classic FD patients [36].

The assessment of renal function that should be carried out includes serum creatinine, cystatin C, eGFR estimated glomerular filtration rate, total urinary protein excretion and urinary albumin excretion. The utility of urine protein/creatinine ratios and eGFR has been established for the staging of chronic kidney disease [37].

For an effective management of underlying kidney pathology, early diagnosis and timely initiation of treatment at a young age are crucial, with the aim to slow down or even reverse glomerular and vascular damage before albuminuria or changes in GFR become overt [38]. Treatment recommendations for FD nephropathy aim at controlling proteinuria to < 0.5 g/day and blood pressure, and at initiating therapy promptly when evidence of kidney involvement occurs. Patients who develop kidney failure should undergo renal replacement therapy (dialysis or kidney transplantation). Angiotensin-converting enzyme inhibitors or angiotensin receptor blockers to reduce proteinuria are also recommended [23].

It is known that GFR constantly declines in patients with FD. Mehta et al. observed a mean yearly decline of − 2.46 − 3.58 ml/min/1.73 m^2^ with ERT compared to the yearly GFR decline of − 1 ml/min/1.7 3 m^2^ in the normal adult population [39, 40].

The potential role of migalastat in the treatment of FD nephropathy has been assessed in two trials. Firstly, in the phase III, double-blind trial comparing migalastat with placebo in ERT-naïve patients with migalastat-amenable mutations (FACETS; *n* = 67), which showed a statistically significant and durable reduction in GL-3 inclusions (proportion of patients with ≥50% reduction in the average number of GL-3 inclusions per interstitial capillary) in favor of migalastat when the analysis included only patients with amenable mutations (mITT population) [17]. In a separate analysis of eight males with amenable mutations enrolled in the FACETS study and for whom paired renal biopsies were available, migalastat was associated with reductions from baseline in mean total volume of GL-3 inclusions per podocyte and mean podocyte volume after 6 months’ treatment [40]. No decrease of renal function was also observed with migalastat during the FACETS trial, with an annualized mean (±SE) change in eGFR from baseline to month 24: − 0.30 ± 0.66 ml/min/1.73 m^2^). On the other hand, there were no significant differences in baseline levels or changes from baseline between study groups for 24-h urinary protein excretion [17].

Secondly, the phase III open-label study (ATTRACT; *n* = 60), demonstrated that migalastat was comparable to ERT (either agalsidase alfa or agalsidase beta) with respect to effects on renal function (annualized changes in GFR from baseline through month 18) in ERT-experienced patients with migalastat-amenable mutations. In addition, the annualized rates of change in eGFR among this group decreased less than the eGFR of historic untreated patients regardless of the baseline levels of urinary protein excretion [15, 41].

In a recent analysis in the subgroup of patients with the classic phenotype treated with migalastat in the FACETS trial, the annualized change in eGFR Chronic Kidney Disease Epidemiology Collaboration (CKD-EPI) was − 0.3 ml/min/1.73 m^2^ compared to historical studies of untreated males reporting an annualized change in eGFR of up to − 12.2 mL/min/1.73m^2^ [24].

A real-life study by Müntze et al., showed a decline in renal function in some patients treated with migalastat. The observation period of only one year after starting chaperone treatment may have been too short to detect a stabilization of the renal function that might require a longer period [25]. In addition, the Authors report that some patients started migalastat and ACE inhibitors or AT1 receptor inhibitors at the same time, thus potentially influencing the GFR reduction independently of the chaperone therapy.

Thus, at the time this overview was written, available data showed that migalastat did not change the renal function decline induced by Fabry disease, in the observation period covered by published studies [15, 17, 24, 25, 40, 41].

### Migalastat in patients with neurological involvement

**Table:** 

Treatment with migalastat can be considered in patients with FD aged ≥16 years with amenable mutations and transient ischemic attack /stroke and/or white matter lesions.	

The first neurological symptoms of FD usually occur in the peripheral nervous system (PNS) as a result of neuronal damage, which contributes to the onset of neuropathic pain, dysesthesias, and sensory deficits, including hearing loss [4]. In the central nervous system (CNS), ischemic stroke and transient ischemic attacks are the most prevalent cerebrovascular complications of FD. The majority of strokes are of lacunar type and mainly due to GL-3 accumulation in the endothelium of small intracranial vessels [42]. Nevertheless, asymptomatic cerebral white matter hyperintensities represent the most common, although aspecific, expression of cerebral involvement in this condition, with a prevalence up to 80% of the cases. Progression of white matter lesions was seen during follow-up irrespective of gender and ERT treatment [43]. The pathophysiology of white matter lesions in FD is complex and not well established; however, these abnormalities seem to be related to stroke, cerebral small vessel dysfunction, cognitive impairment, and motor abnormalities [42, 44].

Since migalastat is able to cross the blood–brain barrier, it might contribute in reducing the occurrence of cerebrovascular events and of white matter lesion load [45]. However, in the phase III ATTRACT study, the low proportion of patients with cerebrovascular events (one in the ERT group and none with migalastat) does not allow to get any firm conclusion [15]. Further studies including a larger population of FD patients with cerebrovascular diseases and/or white matter lesions are necessary to assess the potential relevance of the drug in limiting the progression of neuronal damage.

### Migalastat in patients with other symptoms

**Table:** 

Treatment with migalastat can be considered in patients with FD aged ≥16 years with amenable mutations with acroparaesthesia, and/or gastrointestinal symptoms, and/or hearing loss.	

Neuropathic pain (also called acroparesthesia) is one of the earliest symptoms and is present in approximately 70% of young FD patients with classic phenotype [46]. Hypoidrosis and heat/cold intolerance with pain crises are frequently reported with a negative effect on patients’ quality of life. Neuropathic pain is probably related to progressive reduction in the density of small myelinated and unmyelinated C fibers in the peripheral somatic and autonomic nervous system, while hypohidrosis is mainly due to both small fiber neuropathy and sweat gland tubules GL3 deposits [21]. Some studies suggest a benefit of ERT on neuropathic pain [47–50]. In Phase III ATTRACT study, patients in migalastat group or in ERT group had stable and comparable scores on the Brief Pain Inventory-Short Form during the study period [15].. Clinical evidence for an improvement of acroparesthesiae and hearing loss is still wanted.

Gastrointestinal symptoms are some of the earliest and most frequent symptoms of FD, being reported in approximately 60 and 50% of children and adults, respectively; they include abdominal pain, bloating, diarrhea, constipation, nausea and vomiting, and are associated with a major worsening of quality of life [4]. In the FACETS trial, migalastat-treated patients demonstrated decreased gastrointestinal symptoms for diarrhea, reflux and indigestion per the Gastrointestinal Symptom Rating Scale [17].

In a subanalysis of the FACETS trial, minimal clinically important differences (MCID) in diarrhea based on the corresponding domain of the patient-reported Gastrointestinal Symptom Rating Scale (GSRS) were evaluated [51]. After 6 months’ treatment, more patients receiving migalastat experienced improvement in diarrhea based on a MCID of 0.33, compared with placebo recipients (43% vs 11%; *p* = 0.02). These findings were consistent also in patients with baseline diarrhea (71% vs 20%; p = 0.02).

FD-associated hearing loss can be progressive or sudden [4], and can be due to different reasons [4]. Hearing loss has been reported in 18–55% of FD patients, and tinnitus in 17–53% [4]. It is important to understand the cause of any hearing impairment prior to treatment initiation; audiometry testing and neurological investigations should therefore be performed at diagnosis and then at regular intervals.

### Migalastat in patients with ERT failure/intolerance

**Table:** 

In a patient aged ≥16 years with amenable mutation already in treatment with ERT, switching to migalastat should be considered in the case of poor response and/or uncontrolled infusion reactions and/or poor compliance to intravenous therapy.	

In the opinion of Authors, and based on the results of the ATTRACT study, given the possibility of oral therapy and its efficacy and safety, switching to migalastat can be considered in: (i) unstable patients with clinical evidence of progression of FD, in particular GI symptoms, cardiac hypertrophy and CNS events, (ii) patients with uncontrolled infusion reactions, (iii) patients with oor compliance to i.v. chronic infusions, as shown in the ATTRACT trial [15, 52].

## Conclusions

Although studies and data on longer-term follow up with migalastat are still emerging, available evidence is consistent in showing that this molecule does represent a suitable therapy for the treatment of FD, in patients aged ≥16 years and with amenable mutations and eGFR 30–90 ml/min/1.73 m^2^. The use of migalastat as an oral option appears to be overall safe, and experience thus far indicates potential for improving quality of life, controlling GI symptoms, stabilizing renal function and reducing cardiac hypertrophy.

Migalastat can be considered either as a first-line therapy – given its efficacy, extensive tissue penetration, convenient oral regimen, and the current limited therapeutic options available – or in patients on ERT who experience side effects, poor compliance to chronic i.v. therapy, or in the case of unstable disease. It should be noted that, until data on longer-term follow up are acquired, careful monitoring of patients treated with migalastat is warranted. Increasing real life clinical use, with increasing number of patients closely followed-up for prolonged periods, will be crucial to gain more experience on migalastat in daily practice.

## Data Availability

Data sharing is not applicable, as no datasets were generated or analyzed during the preparation of this article.

## Abbreviations

AE: Adverse events
CNS: Central nervous system
ECG: Electrocardiogram
eGFR: estimated glomerular filtration rate
ERT: Enzyme replacement therapy
FD: Fabry disease
GI: Gastrointestinal
GL3: Globotriaosylceramide
GLA: Galactosidase alpha
GSRS: Gastrointestinal Symptom Rating Scale
HEK: Human Embryonic Kidney
i.v.: intravenous
ITT: Intent-to-treat
KDIGO: Kidney Disease Improving Global Outcomes
KIC: Kidney interstitial capillary
LVH: Left ventricular hypertrophy
LVMi: Left ventricular mass index
MCID: Minimal clinically important difference
MRI: Magnetic resonance imaging
PNS: Peripheral nervous system
QoL: Quality of life
TIA: Transient ischemic attack
